# Role of Cholinergic Anti-Inflammatory Pathway in Protecting Sepsis-Induced Acute Lung Injury through Regulation of the Conventional Dendritic Cells

**DOI:** 10.1155/2022/1474891

**Published:** 2022-01-27

**Authors:** Ruiting Li, Xuemei Hu, Huibin Chen, Yue Zhao, Xuehui Gao, Yin Yuan, Huiling Guo, Haiyan Huang, Xiaojing Zou, Hong Qi, Hong Liu, You Shang

**Affiliations:** ^1^Department of Critical Care Medicine, Institute of Anesthesia and Critical Care Medicine, Union Hospital, Tongji Medical College, Huazhong University of Science and Technology, Wuhan, Hubei 430022, China; ^2^Department of Nephrology, Taihe Hospital, Hubei University of Medicine, Shiyan, Hubei Province 442000, China; ^3^Department of Critical Care Medicine, Taihe Hospital, Hubei University of Medicine, Shiyan, Hubei Province 442000, China; ^4^Department of Critical Care Medicine, Jin Yin-tan Hospital, Wuhan, Hubei 430048, China

## Abstract

**Background:**

The cholinergic anti-inflammatory pathway connects the immune response system and the nervous system via the vagus nerve. The key regulatory receptor is the *α*7-subtype of the nicotinic acetylcholine receptor (*α*7nAChR). Cholinergic anti-inflammatory pathway has been proved to be effective in suppressing the inflammation responses in acute lung injury (ALI). Dendritic cells (DCs), the important antigen-presenting cells, also express the *α*7nAChR. Past studies have indicated that reducing the quantity of mature conventional DCs and inhibiting the maturation of pulmonary DCs may prove effective for the treatment of ALI. However, the effects of cholinergic anti-inflammatory pathway on maturation, function, and quantity of DCs and conventional DCs in ALI remain unclear.

**Objective:**

It was hypothesized that cholinergic anti-inflammatory pathway may inhibit the inflammatory response of ALI by regulating maturation, phenotype, and quantity of DCs and conventional DCs.

**Methods:**

GTS-21 (GTS-21 dihydrochloride), an *α*7nAchR agonist, was prophylactically administered in sepsis-induced ALI mouse model and LPS-primed bone marrow-derived dendritic cells. The effects of GTS-21 were observed with respect to maturation, phenotype, and quantity of DCs, conventional DCs, and conventional DCs2 (type 2 conventional DCs) and the release of DC-related proinflammatory cytokines *in vivo* and *in vitro*.

**Results:**

The results of the present study revealed that GTS-21 treatment decreased the maturation of DCs and the production of DC-related proinflammatory cytokines *in vitro* and in sepsis-induced ALI mouse model; it reduced the quantity of CD11c^+^MHCII^+^ conventional DCs and CD11c^+^CD11b^+^ conventional DCs2 *in vivo* experiment.

**Conclusions:**

Cholinergic anti-inflammatory pathway contributes to the reduction in the inflammatory response in ALI by regulating maturation, phenotype, and quantity of DCs, conventional DCs, and conventional DCs2.

## 1. Introduction

Acute lung injury (ALI) and acute respiratory distress syndrome (ARDS) are common complications associated with both general and acute pulmonary diseases and are characterized by interstitial pulmonary edema, destruction of the alveolar-capillary barrier of the lungs, and excessive inflammatory response in the lung tissues [1]. Apart from the direct intrapulmonary factors, sepsis is the major extrapulmonary risk factor associated with ALI/ARDS. In more than half of the patients, sepsis progresses into ALI/ARDS, but there is not much clarity on the pathological mechanisms of sepsis-induced ALI/ARDS [2].

Both innate and adaptive immune response participate in the pathological process of ALI/ARDS [3, 4]. Innate immune response is the first line of defense when lung injury occurs and plays a key role in regulating its excessive inflammatory response. In addition, adaptive immune also plays an important role in inflammatory processes through regulating T cells, dendritic cells (DCs), and other immune regulatory cells [3, 4]. DCs, T cells, and neutrophils in turn play key roles in the advancement of ALI through integrating innate and adaptive immunity, release of inflammatory cytokines, and activation of pro-inflammatory signaling pathway thereby controlling pulmonary inflammation [3, 4]. Hence, the inhibition of the pro-inflammatory pathway and the activation of the anti-inflammatory pathway of innate and adaptive immune response are crucial for the survival of patients [3, 4]. The cholinergic anti-inflammatory pathway (CAP) establishes connection between the immune response and the nervous system through the vagus nerve. The key regulatory receptor is the *α*7-subtype of the nicotinic acetylcholine receptor (*α*7nAChR), which is localized on the surface of immune cells. CAP has been proved to be effective in many pathologic processes including suppressing inflammation responses and improving endotoxemia, sepsis, cancer, and ischemia-reperfusion-induced ALI [5, 6]. *α*7nAChR signaling also have other biological effects, including antifibrosis, antitumor and anti-inflammation in chronic respiratory diseases [6, 7]. Considering the significant effect of CAP on the pathological process of ALI, *α*7nAChR has been considered as a potential intervention target for inhibiting excessive inflammatory response of ALI.

*α*7nAChR is also distributed on the surface of cells of the immune system, in addition to that of the nervous system. Previous studies have reported that *α*7nAChR is also expressed on the surface of DCs and plays a key role in the inhibition of immune response in DCs and T cells in the context of the CAP [8, 9]. DCs, as important antigen-presenting cells (APCs), also serve as immune cells, playing the key role in immune response priming, and participate in the pathological process of ALI/ARDS [10–12].

Previous studies have indicated that reducing the quantity of mature conventional DCs (cDCs) and inhibiting the maturation of pulmonary DCs may prove effective for the treatment of ALI [13, 14]. CD11c^+^ DCs were found to be the key protecting cells in transfusion-related acute lung injury (TRALI), and this protection occurs via IL-10. Depletion of CD11c^+^ DCs in vivo increased the susceptibility to induce TRALI [14, 15]. However, the effects of CAP on the maturation and function of DCs and cDCs in ALI/ARDS remain unclear.

Therefore, it was hypothesized that the activation of CAP may inhibit the excessive inflammatory response of ALI by mediating maturation, phenotype, and quantity of DCs and cDCs. This may have been proved to be a new intervention in the treatment of ALI.

## 2. Material and Methods

### 2.1. Animals

SPF C57BL/J6 male mice (aged 6–8 weeks, weight 20 ± 2 g) were purchased from the Experimental Animal Centre of Hubei province (Wuhan, China). All experimental procedures were performed in compliance with the National Institutes of Health Guidelines and were approved by Huazhong University of Science and Technology (Wuhan, China) experimental animal ethics committee.

### 2.2. Establishment of ALI Mouse Model and Administration of GTS-21

All experimental mice were randomly divided into 4 groups (*n* = 4–6 mice per group): control group; sepsis-induced ALI group; GTS-21 (GTS-21 dihydrochloride, a *α*7nAchR agonist; Abcam, Cambridge, UK) positive control group; and ALI-treated by GTS-21 group. No differences in the food intake or body weight were observed among the groups. In order to induce ALI, the mice in the ALI group and ALI treated by GTS-21 group were administered with the intraperitoneal (i.p.) injection of 200 *μ*L sterile phosphate-buffered saline (PBS), which contained 15 mg/kg lipopolysaccharide (LPS, Sigma Aldrich, St. Louis, MO, USA). The control and GTS-21-positive control groups were administered with only 200 *μ*L of sterile PBS at the same time points. In order to activate the CAP, the positive control and treatment groups prophylactically received an i.p. injection of 4 mg/kg GTS-21, diluted in 200 *μ*L of PBS, 30 min before the administration of LPS [13]. All the mice were sacrificed after blood and the broncho-alveolar lavage fluid (BALF) were collected, 24 h after the LPS injection. This was followed by the removal of the lungs and spleens, which were temporarily stored in RPMI-1640 supplemented with 10% fetal bovine serum (FBS) (HyClone; Logan, UT, USA) at 4°C for further investigation.

### 2.3. Collection of BALF

The lungs were flushed three times with 0.5 mL PBS via a tracheal cannula to collect BALF. The BALF was centrifuged, then the supernatant was collected for the subsequent analysis of cytokine levels [13].

### 2.4. Preparation of Bone Marrow-Derived DCs (BMDCs)

The bone marrow was extracted from the femur bones of C57BL/J6 mice (age: 5–8 weeks). On day 0, bone marrow mononuclear cells (MNCs) were seeded at 1–2 × 10^6^ cells/mL in 15 mL RPMI-1640 that contained 10 ng/mL granulocyte-macrophage colony-stimulating factor (GM-CSF) (PeproTech, Rocky Hill, NJ, USA) and 10 ng/mL recombinant mouse IL-4 (rmIL-4) (PeproTech, Rocky Hill, NJ, USA), then they were cultured in an incubator under the following conditions: 37°C and 5% CO_2_. On days 3 and 5, 10 mL freshly prepared RPMI-1640 containing 10 ng/mL rmIL-4 and GM-CSF were added to the plates to change the culture medium. On day 7, the BMDCs were collected and resuspended in RPMI-1640 medium at a density of 1–2 × 10^6^ cells/mL; the purity of the immature DCs in in the samples was at least 90%. The BMDCs were then incubated with or without LPS (100 ng/mL) and GTS-21 (100 mM) for 24 h [13]. The BMDC culture supernatants were collected for subsequent enzyme-linked immunosorbent assay (ELISA) assay. The BMDCs were frozen at -80°C for further analysis.

### 2.5. ELISA

The levels of cytokines [interleukin-6 (IL-6), tumor necrosis factor-alpha (TNF-*α*), IL-12p40, IL-18, IL-1*β*, and high mobility group box 1 (HMGB1)], secreted by DCs, were measured by ELISA, according to manufacturer's instructions. The ELISA detection kits of IL-6, TNF-*α*, IL-12p40, IL-18, and IL-1*β* were purchased from eBioscience (San Diego, CA, USA), and the HMGB1 ELISA kit was purchased from LifeSpan BioSciences, Inc. (Seattle, Wash).

### 2.6. Flow Cytometric Analysis

Lung single-cell suspensions and BMDC suspensions collected from different groups were stained with PE anti-mouse CD11c (eBioscience, San Diego, CA, USA) and APC-Cy7 anti-mouse F4/80 (Biolegend Inc., San Diego, CA, USA). DCs were marked as CD11c^+^ and F4/80^−^ cells. This was followed by analysis of the phenotype and the maturation of DCs using FITC anti-mouse CD80, FITC anti-mouse major histocompatibility complex II (MHCII), FITC anti-mouse CD40 (eBioscience, San Diego, CA, USA), and APC anti-mouse CD86 (Biolegend Inc., San Diego, CA, USA) stains. Among them, cDCs were marked as CD11c^+^MHCII^+^ double-positive cells. In addition, the cDCs2 (type 2 cDCs) in lung and spleen MNCs were stained as CD11c^+^CD11b^+^ double-positive cells using PE anti-mouse CD11c and APC anti-mouse CD11b (Biolegend Inc., San Diego, CA, USA). All the cells, after being washed with PBS, were analyzed using Flow Cytometer (FACSAria™ III, BD Biosciences, USA). The FlowJo software (FlowJo LLC, Ashland, Ore) was used to analyze the data.

### 2.7. Statistical Analysis

All results are presented as mean ± standard deviation (SD). All experiments were repeated more than thrice (*n* = 4‐6 mice/group). Presented data are one representative experiment of three repeats. Statistical analysis was performed using the one-way ANOVA. Data were analyzed using the SPSS 22.0 software (IBM SPSS, Chicago, IL, USA). A *p* value of <0.05 was considered significant.

## 3. Results

### 3.1. CAP Inhibited the Maturation of DCs in Sepsis-Induced ALI Mouse Model

**Figure 1:**
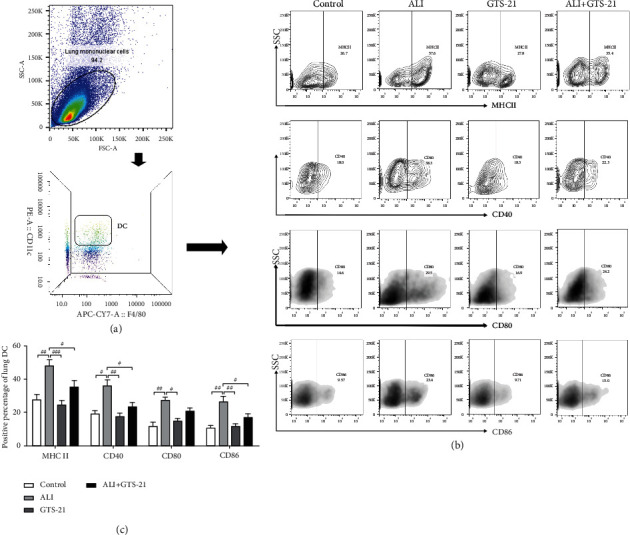
GTS-21 administration inhibited the maturation of DCs in the lungs of ALI induced by sepsis. (a) Flow cytometry analysis was used to examine the CD11c^+^ F4/80^−^ lung DCs isolated from lung MNCs of mice. (b, c) Detecting the percentages of MHCII-, CD40-, CD80-, and CD86-positive expression on the surface of lung DCs. *n* = 4–6 mice/group. All data are shown as mean ± SD. ^#^*p* < 0.05, ^##^*p* < 0.01, ^###^*p* < 0.001.

To assess the role of CAP on differentiation and maturation of DCs in sepsis-induced ALI mouse model (i.p. injected LPS), GTS-21, the *α*7nAchR agonist, was i.p. injected 30 min before LPS intervention in order to activate the CAP. After 24 h of the administration of LPS, suspensions of lung MNCs were prepared. Flow Cytometry was used to analyze the expression of antigen-presenting molecule MHCII and costimulatory molecules (CD80, CD86, and CD40) on surface of CD11c^+^F4/80^−^ DCs (Figure 1(a)). Mature differentiation of DCs was characterized by the expressions of CD80, CD86, CD40, and MHCII on their surface [16]. Lung MNCs were first stained with PE anti-mouse CD11c and APC-Cy7 anti-mouse F4/80, and the DCs were detected as CD11c^+^F4/80^−^ MNCs (Figure 1(a)). It was found that the percentages of MHCII, CD40, CD80, and CD86 on surface of DCs obviously increased in the sepsis-induced ALI group when compared to the control and GTS-21-positive control mice group, and the increase in MHCII, CD40, and CD86 was reduced through GTS-21 intervention (Figures 1(b) and 1(c)). Therefore, CAP has the potential to inhibit mature differentiation of DCs in sepsis-induced ALI mouse model.

### 3.2. Downregulation of the Levels of DC-Related Pro-inflammatory Cytokines in ALI Mouse Model through GTS-21 Treatment

**Figure 2:**
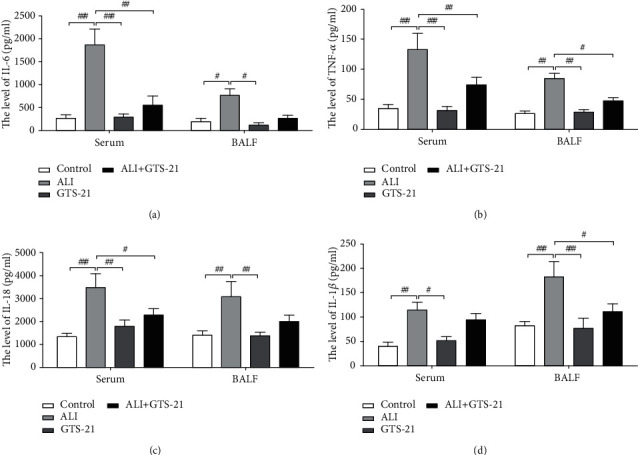
GTS-21 administration reduced the releases of DC-related proinflammatory cytokines in the serum and BALF of sepsis-induced ALI mice. (a–d) The productions of cytokines IL-6, TNF-*α*, IL-18, and IL-1*β* in the serum and BALF were measured by ELISA. *n* = 4–6 mice/group. All data are shown as mean ± SD. ^#^*p* < 0.05, ^##^*p* < 0.01, ^###^*p* < 0.001.

To examine the effect of CAP on DC-related pro-inflammatory cytokines, the levels of IL-6, TNF-*α*, IL-18, and IL-1*β* in the serum and BALF of different group mice were detected. The release of TNF-*α*, IL-6, IL-18, and IL-1*β* in the serum along with BALF was observed to be significantly higher in the ALI groups when compared to that in the control and GTS-21-positive control mice group. On the other hand, the administration of GTS-21 caused a decrease in the level of cytokines TNF-*α*, IL-6, and IL-18 in the serum and the release of cytokines TNF-*α* and IL-1*β* in BALF of ALI mice (Figures 2(a)–2(d)). In the previous studies conducted by the investigators, it was found that GTS-21 was involved in the downregulation of the expression and the release of two other DC-related cytokines (IL-12p40 and HMGB1) in ALI [13]. Taking into consideration the findings of the current research and that of the previously conducted study, it can be inferred that CAP is responsible for a significant decrease in the levels of these pro-inflammatory cytokines released by DCs *in vivo*.

### 3.3. CAP Reduced the Quantity of cDCs and cDCs2 in ALI Mouse Model

**Figure 3:**
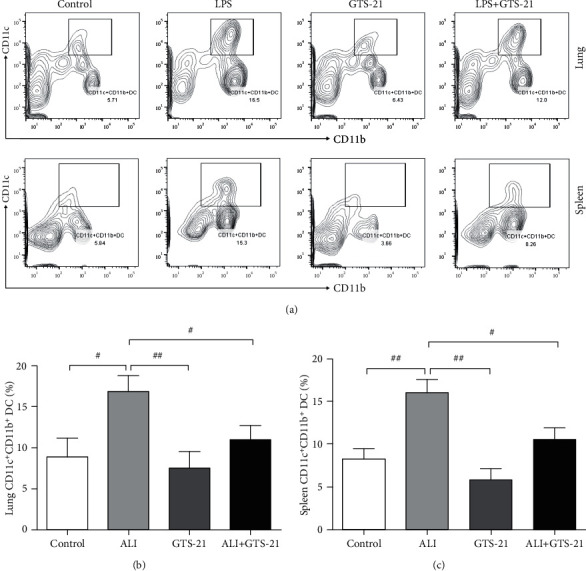
GTS-21 treatment reduced the number of cDCs2 in the lungs and spleens of sepsis-induced ALI mice. (a) Flow cytometry analysis was used to examine the CD11c^+^CD11b^+^ cDCs2 isolated from the lung and spleen MNCs of mice. (b, c) The relative percentages of lung and spleen CD11c^+^CD11b^+^ cells in different groups. *n* = 4–6 mice/group. All data are shown as mean ± SD. ^#^*p* < 0.05, ^##^*p* < 0.01, ^###^*p* < 0.001.

The DCs have two subsets: plasmacytoid DCs (pDCs) and cDCs. cDCs, the predominating DC population, are preferentially localized in the lung interstitium and play a significant role in systematic inflammatory response [17]. cDCs (CD11c^+^MHCII^+^) in the lung are represented by CD11c^+^CD103^+^ DCs (type 1 cDCs, cDCs1) and CD11c^+^CD11b^+^ DCs (cDCs2), and cDCs2 in the lungs were identified as CD11b^+^. In the spleen, cDCs are also classified into the subsets according to the CD11b marker expression [17–19]. It was recently reported that the population of CD11c^+^CD11b^+^ DCs (cDCs2) sense pathogens, produce proinflammatory cytokines, and drive naive CD4^+^ T cells toward distinct effector subsets [20]. cDCs were marked as CD11c^+^MHCII^+^, and the positive percentage of MHCII not only represented the maturation of DCs but also represented the total population of cDCs. As depicted in the part of MHCII in Figures 1(b) and 1(c), the percentage of cDCs in the lung tissues of the ALI group showed a significant increase when compared to those from the control mice, and GTS-21 treatment caused a significant downregulation of the percentage of cDCs compared with that in the ALI mice. In order to examine the role of GTS-21 on cDCs2, the CD11c^+^CD11b^+^ double-positive DCs were stained and separated from lung and spleen MNCs of different groups. The percentages of cDCs2 in the lung and spleen were markedly higher in the ALI group as compared to that in the control and GTS-21-positive control group. GTS-21 administration downregulated the percentages of pulmonary and splenic cDCs2 when compared with that in the ALI group (Figures 3(a)–3(c)). These findings suggested that CAP activation can result in the downregulation of the number of cDCs and cDCs2 in ALI.

### 3.4. GTS-21 Inhibited the Maturation of DCs *In Vitro*

**Figure 4:**
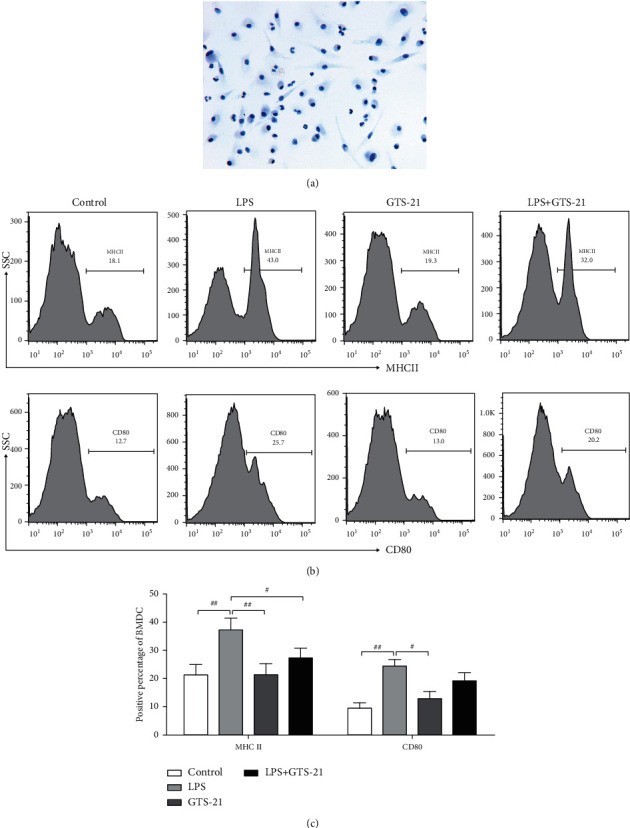
GTS-21 treatment weakened the mature differentiation of DCs *in vitro*. (a) The morphology of BMDCs on days 7 of culture (400x magnification). (b, c) Flow cytometry analysis was used to examine the percentages of MHCII- and CD80-positive expression on the surface of CD11c^+^ F4/80^−^ BMDCs. All data are shown as mean ± SD (*n* = 4–6 mice/group). ^#^*p* < 0.05, ^##^*p* < 0.01, ^###^*p* < 0.001.

To analyze the effect of CAP on mature differentiation of DCs *in vitro*, BMDCs were stimulated with LPS to induce maturation, and GTS-21 was added to observe whether it inhibited the maturation process of BMDCs.  Figure 4(a) depicts the morphology of BMDCs (numerous extended dendrites in different directions from the cell body). Flow Cytometry was used to analyze the expression of MHCII and CD80 on the surface of DCs. It was found that the percentages of MHCII and CD80-positive expression were markedly elevated in the case of LPS-primed BMDCs group in comparison to that in the control BMDCs group. On the other hand, GTS-21 significantly inhibited the expression of MHCII on LPS-primed BMDCs (Figures 4(b) and 4(c)). The obtained data suggested that CAP inhibited the differentiation of DCs into mature cells *in vitro*.

### 3.5. GTS-21 Downregulated the Levels of DC-Related Pro-inflammatory Cytokines *In Vitro*

**Figure 5:**
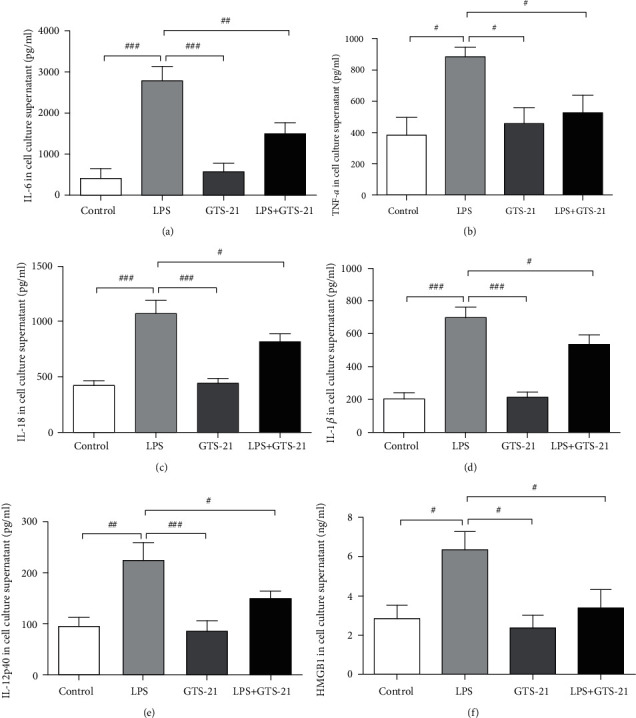
GTS-21 administration reduced DC-related proinflammatory cytokine levels *in vitro*. (a–d) The productions of cytokines IL-6, TNF-*α*, IL-18, IL-1*β*, IL-12p40, and HMGB1 in BMDC culture supernatant were measured by ELISA. All data are shown as mean ± SD (*n* = 4–6 mice/group). ^#^*p* < 0.05, ^##^*p* < 0.01, ^###^*p* < 0.001.

ELISA analysis was performed to analyze the effect of GTS-21 on the release of DC-related pro-inflammatory cytokines from LPS-primed BMDCs for measuring the levels of IL-6, TNF-*α*, IL-18, IL-1*β,*IL-12p40, and HMGB1 in the BMDC culture supernatant. It was observed that the production of IL-6, TNF-*α*, IL-18, IL-1*β*, IL-12p40, and HMGB1 was markedly upregulated due to LPS stimulation when compared to that in the control group. On the other hand, GTS-21 treatment markedly decreased the production of the IL-6, TNF-*α*, IL-18, IL-1*β*, IL-12p40, and HMGB1 cytokines in the BMDC culture supernatant (Figures 5(a)–5(f)). These results suggested that CAP has the potential to inhibit the production of pro-inflammatory cytokines from DCs *in vitro*.

## 4. Discussion

ALI is characterized by inflammatory cell infiltration and recruitment to the injured lung interstitium. Among them, DCs are the APCs that play a significant role in inflammatory response in the case of sepsis-induced lung injury. Numerous studies have suggested that by adopting different ways to decrease recruitment of activated DCs and cDCs in the lung may reduce the incidences of lung injury [11, 12]. In our present study, it was found that GTS-21 treatment decreased the maturation of DCs and the release of DC-related pro-inflammatory cytokines *in vitro* and in sepsis-induced ALI mouse model and reduced the quantity of CD11c^+^MHCII^+^ cDCs and CD11c^+^CD11b^+^ cDCs2 *in vivo* experiment. Thus, it can be inferred that the activation of CAP may cause decreased inflammatory response in ALI through the regulation of maturation, phenotype, and quantity of DCs, cDCs, and cDCs2.

The acute inflammatory response in ALI not only includes the activation of innate and adaptive immune but also certain specific activities of the autonomic nervous system. The vagus nerve is considered to perform anti-inflammatory functions, by inhibiting the release of pro-inflammatory cytokines and protecting the lung from pathological injury; this anti-inflammatory function of the vagus nerve is termed as CAP [21]. It has been confirmed that CAP protects the lungs from infection and sepsis. In this signaling pathway, *α*7nAChR is a key target receptor that can attenuate the production of pro-inflammatory mediators from inflammatory cells, such as macrophages, DCs and neutrophils, at the same time as expressing itself on the surface of these inflammatory cells [9, 22]. Recent studies suggested that *α*7nAChR inhibited the activation of downstream inflammasomes, decreased the secretion of pro-inflammatory cytokines and chemokines from macrophages, which play an important role in regulating the progression of lung injury, and reduced lung inflammatory injury [23, 24]. *α*7nAchR also can reduce the expression of Caspase-1 and IL-1*β* associated with pyroptosis, a programmed cell death pathway which plays an important role in the process of lung injury [23]. A number of studies have shown that the activation of CAP inhibited differentiation and maturation of DCs, reduced DC-associated cytokine release, and ameliorated the inflammatory responses in the body [9]. However, the relationship between CAP and DCs in ALI remains unclear. In studies conducted by the investigators, it was found that the anti-inflammatory effect of the CAP agonist GTS-21 on DCs in sepsis-induced ALI: GTS-21 significantly inhibited the expression of MHCII, CD40, and CD86 on the surface of DCs and also the release of DC-related pro-inflammatory cytokines (IL-6, TNF-*α*, IL-18, IL-1*β*, IL-12p40, and HMGB1).

DCs through identification of the antigens of the pathogens present antigens to activate T cells and initiate antigen-specific cellular immune responses, which are associated with many diseases, and play a critical role in the pathological process of ALI. In mouse and human beings, there are different types of DCs with different phenotypes, functions, and localizations that form an immune system, which is distributed across all organs of the body and is responsible for immune-surveillance [19, 25, 26]. DCs can be classified into two main lineages, cDCs and pDCs, which are crucial for the priming phase of the immune response. A number of studies have reported that marked increase in mature cDCs plays a key pro-inflammatory role in lung injury. As ALI progresses, cDCs express MHCII and CD80, which are rapidly accumulated in the lung interstitium, resulting in the production of a range of inflammatory mediators [11, 12, 26, 27]. These findings are consistent with the findings of the current study. Moreover, the data obtained in the current study also confirmed immunomodulatory effects of CAP on cDCs in pathological process of ALI using GTS-21. GTS-21 caused significant reduction in the quantity of CD11c^+^MHCII^+^ cDCs in LPS-induced mice model and in *in vitro* experiment. The data suggested that the anti-inflammatory activity of the CAP in ALI can be attributed to regulation of the number and accumulation of cDCs.

cDCs can also be further divided into two main subsets: cDCs1 and cDCs2. In mouse, the cDCs1 are characterized by the expression of CD103 and cDCs2 phenotype being CD11c^+^MHCII^+^CD11b^+^. The subset cDCs1 perform a specialized function of recognizing exogenous antigens and activating the CD8^+^ T cell immune response, whereas the other subset cDCs2 is primarily responsible for presenting endogenous antigens to CD4^+^ T cells and stimulating the polarization in these cells [28–30]. CD11c^+^CD11b^+^ cDCs are the most abundant DCs in the lymphoid organs and are also found in the spleen, skin, and lung tissues. Previous studies have demonstrated that CD11c^+^CD11b^+^ cDCs2 derived Th (T help cell) 17, Th2-immune responses in asthma, lung emphysema, and COPD [31–33]. CD11c^+^CD11b^+^ cDCs2 also produce pro-inflammatory cytokines such as IL-6 and IL-23 [33, 34]. Li et al. [35] reported that when CD11c^+^CD11b^+^ cDCs2 were cocultured with T cells or LPS, they not only displayed immunoregulatory characteristics but also played a crucial role in the induction of immune tolerance of experimental autoimmune encephalomyelitis (EAE). However, the role of CD11c^+^CD11b^+^ cDCs2 in ALI remains unclear, in particular, the relationship between cDCs2 and CAP. In the current study, the number of CD11c^+^CD11b^+^ cDCs2 in the spleen and lung was found be upregulated in the LPS-induced ALI mice. In addition, the results also demonstrated that GTS-21, the CAP agonist, downregulated the phenotypic expression of cDCs2 in ALI mice model. Of course, there are still some limitations in the present study. We obtained these results only with the intervention of *α*7nAchR agonists, without the use of inhibitors or downregulation of the expression of this receptor to verify the feasibility of these mechanisms, which is what we will study next.

## 5. Conclusion

In this study, we found that CAP could reduce the maturation of DCs and the production of DC-related pro-inflammatory cytokines both in vitro and in vivo and decreased the quantity of cDCs and cDCs2 in vivo. Therefore, CAP played a crucial role in the pathological process of sepsis-induced ALI through regulating the number of DCs, cDCs, and cDCs2 and also their differentiation into mature cells.

## Data Availability

Access to data is restricted.

## Abbreviations

*α*7nAChR: *α*7-subtype of the nicotinic acetylcholine receptor
ALI: Acute lung injury
ARDS: Acute respiratory distress syndrome
DCs: Dendritic cells
APCs: Antigen-presenting cells
cDCs: Conventional DCs
GTS-21: GTS-21 dihydrochloride
i.p.: Intraperitoneally
LPS: Lipopolysaccharide
BMDCs: Marrow-derived dendritic cells
CAP: Cholinergic anti-inflammatory pathway
cDCs: Conventional dendritic cells
BALF: Bronchoalveolar lavage fluid
RPMI: Roswell Park Memorial Institute
FBS: Fetal bovine serum
PBS: Phosphate-buffered saline
MNCs: Mononuclear cells
GM-CSF: Granulocyte-macrophage colony-stimulating factor
rmIL-4: Recombinant mouse IL-4
ELISA: Enzyme-linked immunosorbent assay
IL: Interleukin
TNF: Tumor necrosis factor
HMGB1: High mobility group box 1
SD: Standard deviation
pDCs: Plasmacytoid dendritic cells
cDCs1: Type 1 conventional dendritic cells
cDCs2: Type 2 conventional dendritic cells
TRALI: Transfusion-related acute lung injury.
